# Estimating Diagnostic Test Accuracies for *Brachyspira hyodysenteriae* Accounting for the Complexities of Population Structure in Food Animals

**DOI:** 10.1371/journal.pone.0098534

**Published:** 2014-06-06

**Authors:** Sonja Hartnack, Christina Nathues, Heiko Nathues, Elisabeth Grosse Beilage, Fraser Iain Lewis

**Affiliations:** 1 Section of Epidemiology, Vetsuisse Faculty, Zurich, Switzerland; 2 Veterinary Public Health Institute, Vetsuisse Faculty, Liebefeld, Switzerland; 3 Department of Production and Population Health, Royal Veterinary College London, London, United Kingdom; 4 Department of Clinical Veterinary Medicine, Vetsuisse Faculty, Zurich, Switzerland; 5 Field Station for Epidemiology, University of Veterinary Medicine Hannover, Foundation, Bakum, Germany; Naval Research Laboratory, United States of America

## Abstract

For swine dysentery, which is caused by *Brachyspira hyodysenteriae* infection and is an economically important disease in intensive pig production systems worldwide, a perfect or error-free diagnostic test (“gold standard”) is not available. In the absence of a gold standard, Bayesian latent class modelling is a well-established methodology for robust diagnostic test evaluation. In contrast to risk factor studies in food animals, where adjustment for within group correlations is both usual and required for good statistical practice, diagnostic test evaluation studies rarely take such clustering aspects into account, which can result in misleading results. The aim of the present study was to estimate test accuracies of a PCR originally designed for use as a confirmatory test, displaying a high diagnostic specificity, and cultural examination for *B. hyodysenteriae*. This estimation was conducted based on results of 239 samples from 103 herds originating from routine diagnostic sampling. Using Bayesian latent class modelling comprising of a hierarchical beta-binomial approach (which allowed prevalence across individual herds to vary as herd level random effect), robust estimates for the sensitivities of PCR and culture, as well as for the specificity of PCR, were obtained. The estimated diagnostic sensitivity of PCR (95% CI) and culture were 73.2% (62.3; 82.9) and 88.6% (74.9; 99.3), respectively. The estimated specificity of the PCR was 96.2% (90.9; 99.8). For test evaluation studies, a Bayesian latent class approach is well suited for addressing the considerable complexities of population structure in food animals.

## Introduction

### Currently used diagnostic tests for *Brachyspira hyodysenteriae*

Swine dysentery (SD) is caused by infection with the bacterium *Brachyspira hyodysenteriae* and imposes major economic losses on intensive pig production systems worldwide [1]. The affected animals, usually growing or finishing pigs, show non-specific clinical symptoms, such as diarrhoea, reduced growth and decreased food conversion which precludes a diagnosis based solely on pathognomonic signs. More specific signs, i.e. large amounts of mucus and often flecks of blood in the faeces, can become visible during the course of the disease, but this is not guaranteed. Finally, sub-clinical disease can occur. The accurate diagnosis of sub-clinical, as well as clinical disease, is a prerequisite for providing efficient treatment procedures, and to support prevention programmes in the field.

Animals in intensive pig production systems are reared in distinct population groups, i.e. herds or farms, and within each group may share multiple genetic and immunologic characteristics. Animals, therefore, from the same population group will likely be more similar to each other than to animals from other groups. In epidemiological terms this may result in over-dispersion, that is, the level of variation in disease prevalence (say) across multiple population groups (e.g. multiple herds or farms) may be far in excess of that allowed by commonly used statistical methods for estimating diagnostic accuracy. Failure to account for such population clustering may lead to highly unreliable results. Moreover, such adjustment for excess variation is common place in other types of epidemiological analyses, e.g. risk factor studies, but as yet rare in diagnostic test evaluation despite identical issues being present.

The development of methods for dealing with the complexity of population structures found in food animal production, such as clustered or hierarchical data, has been described as the single most important advancement [2] for animal health researchers. To ensure success, animal disease prevention programs, typically organized at a national level, need to consider the inherent complexity of the population structure, rather than simply treating the population as an assemblage of individual and unrelated or independent animals. With regard to data from diagnostic test studies, this also holds true for laboratories which receive samples for large-scale testing from national animal populations. The necessity to consider variation of test accuracies at farm level, where test errors might be clustered, has been stated by Donald and co-workers [3], [4]. The ultimate diagnosis of SD should be based on the isolation of the causative agent, but this procedure is extremely laborious, time-consuming and expensive, as the bacteria are fastidious and grow slowly. Another disadvantage of isolation is the fact that both confirmation and differentiation between *B. hyodysenteriae*, and other less- or non-pathogenic *Brachyspira* spp., are dependent upon colony morphology, pattern and intensity of haemolysis and other growth characteristics, which might be very similar among different subspecies [5]. Finally, isolation often fails to recover organisms when infected pigs have been submitted to antimicrobial therapy prior to sampling. This inevitably leads to a decreased diagnostic sensitivity of cultural isolation, and as such, cultural isolation cannot be assumed to be a perfect gold standard for diagnostic test evaluation studies.

Polymerase chain reaction (PCR) is increasingly used for diagnostic purposes, and produces more specific and sensitive results in the detection of *B. hyodysenteriae* when compared with other diagnostic methods [6], [7]. Others found PCR for *B. hyodysenteriae* being less sensitive than culture [8], underlining that none of these tests perform as a perfect gold standard, that is, completely error-free (display 100% diagnostic sensitivity and specificity). A number of false negative test results due to a less than perfect sensitivity, and thus falsely classified animals may contribute to the spread of a contagious agent. False positive test results may lead to welfare and ethical issues if healthy animals are subsequently culled. Reliable information about test accuracies, including information about how well a diagnostic test performs in a defined animal population, is crucial for efficient prevention and control programs.

Generally speaking, for diagnostic test evaluation, the difficulty of obtaining robust estimates for diagnostic sensitivity and specificity by comparing a new diagnostic test with an established standard test, which presumably is not a perfect gold standard, is due to the uncertainty associated with results from the standard test. If for example a highly sensitive PCR is compared with culture as a standard test, which might be highly specific but less sensitive, a number of samples might be correctly classified as being positive by the PCR, but – due to a lower sensitivity – be falsely classified as negative by culture. In this case, if the diagnostic specificity of the PCR is derived by determining the quotient of PCR negative test results divided by the number of culture negative results, then the diagnostic specificity of the PCR might be biased, being too low.

### Using latent class models to estimate test accuracies

Statistical methods have been developed to deal with situations of test evaluation where no gold standard test is readily available. Seminal work was done by Hui and Walter – [9] after whom the Hui-Walter paradigm was named (reviewed by [10] and [11] with regard to the underlying assumptions with regard to the characteristics of diagnostic test data). The term “latent” here refers to the fact that the dichotomized test results contain, only latently, statistical information about the parameters of interest i.e. sensitivities, specificities, and prevalences. These parameters are not directly observed but can be extracted from the data via an appropriate statistical model. There are many extensions to the original Hui-Walter paradigm. Most notably, models for the inclusion of conditional dependencies between diagnostic test accuracies have been developed [12]–[18]. Furthermore, latent class models are now recommended in the Standard Operating Procedures (SOP) of the OIE for validation and certification of diagnostic assays [19].

The key caveat in using latent class or variable models is that the parameter estimates obtained will be, to a greater or lesser extent, dependent upon the model used to extract the latent information from the observed data. Hence, particular attention must be paid when selecting an appropriate model formulation – one that best matches the type and structure of the study data. In respect of food animal production the complexity of the population structure from which study data is sampled is obviously of major importance. To date in veterinary medicine, diagnostic test accuracy studies rarely perform data analysis with regard to potential clustering, with only a few but notable exceptions [20]–[23].

The aim of the present study was to estimate test accuracies of a PCR and cultural testing for *B. hyodysenteriae* on 239 samples from 103 herds originating from routine diagnostic sampling at a laboratory in Bakum, Germany. Given the number of different population groups included in the study data the resulting accuracy estimates could then be considered as broadly applicable to the national swine population. The PCR has been designed to be used as a confirmatory test displaying a high diagnostic specificity.

## Material and Methods

### Samples

Faecal samples (n = 239) originating from 103 herds that had been submitted to the Field Station for Epidemiology in Bakum for the purpose of routine diagnostics due to diarrhoea in pigs were included in this study. These samples arrived in the laboratory during 2007 and 2011, and they were all examined for *B. hyodysenteriae* by both, PCR and cultural testing.

Upon arrival in the laboratory faecal samples were carefully homogenized and each time one aliquot of 200 mg was transferred to a microtube for storage at −20°C. From the remaining faeces a swab was obtained, transferred into a container with transport medium and subsequently shipped to the Institute of Bacteriology, University of Veterinary Medicine Hannover for cultural testing.

PCR was exactly performed as previously described [24]. This PCR had been designed to be used as a confirmatory test displaying a high diagnostic specificity in detecting *B. hyodysenteriae*, *B. pilosicoli* and/or *Lawsonia intracellularis*. For cultural isolation swabs were streaked on Columbia blood agar plates (Oxoid, Germany) and on TSA-plates (Trypticase Soy Agar) containing 0.1% yeast extract, 6 µg/ml vancomycin, 6.25 µg/ml colistin, 12.5 µg/µl rifampicin, 15.25 µg/ml spiramycin, 200 µg/ml spectinomycin, and 5% bovine blood. These were incubated anaerobically (AnaeroJar with AnaeroGen, Oxoid) at 42°C for six consecutive days. Suspected growth of *Brachyspira spp*. was confirmed and species were identified using nox-RFLP as described previously [25].

The raw data of the 239 faecal samples tested in parallel by PCR and culture are shown in table 1.

**Table 1:** Dichotomized diagnostic test results of 239 porcine faecal samples tested in parallel by culture and PCR for *Brachyspira hyodysenteriae.*

		Culture	
		+	−
PCR	+	52	18
	−	11	158

### Statistical analysis

A Bayesian latent class analysis was performed to obtain robust estimates for test accuracies of PCR and culture. It was assumed, as is standard practice, that sensitivities and specificities of the tests are constant across all animals. With the exception of culture, where the specificity was set to 1, uninformative (“flat”) prior distributions on key model parameters were utilized (prior beta distribution (1,1) for the sensitivities of PCR and culture, for the specificity of PCR, and prior uniform distribution (−0.01,0.01) for conditional test dependence between the two sensitivities). A sensitivity analysis for three different beta priors was conducted utilising Betabuster (http://www.epi.ucdavis.edu/diagnostictests/betabuster.html) to define the two shape parameters. The priors have been chosen as input information “to be 95% sure that the parameter of interest is greater than 0.3, 0.4 and 0.5 and the mode is at 0.6, 0.75 and 0.95” respectively. The model parameters were estimated using a hierarchical beta-binomial approach which allowed the prevalences for individual population groups (farms) to vary as a farm level random effect. In other words, we allow different farms to have potentially very different prevalences subject to the condition that the overall distribution of farms prevalences should follow a beta probability distribution (which is very flexible in terms of shape). Gamma distributed priors of (5, 0.01) were used to model the shape parameters of this beta distribution (describing the population level prevalence – i.e. the distribution of within farm prevalences across all farms). Three different gamma priors were tested in a sensitivity analysis.

The effect of conditional dependency between test results, specifically two-way covariance between test sensitivities, was assessed by formal model selection using DIC (deviance information criterion) as the goodness-of-fit criterion, with lower values indicating a better model fit [26]. All models were fitted using Markov chain Monte Carlo estimation through JAGS software (Just Another Gibbs Sampler) (http://mcmc-jags.sourceforge.net/) version 3.3.0. Good standard practice was followed doing MCMC running four chains independently for 500,000 iterations after a burn-in of 25,000 iterations, and thinning of (10), thus resulting 10,000 values to derive the posterior means. Code for the final model is presented in code S1. The output was analysed with the package coda [27] within the software R (http://www.r-project.org/) version 2.15.2. Gelman-Rubin statistics was used to assess mixing [28].

## Results

**Figure 1:**
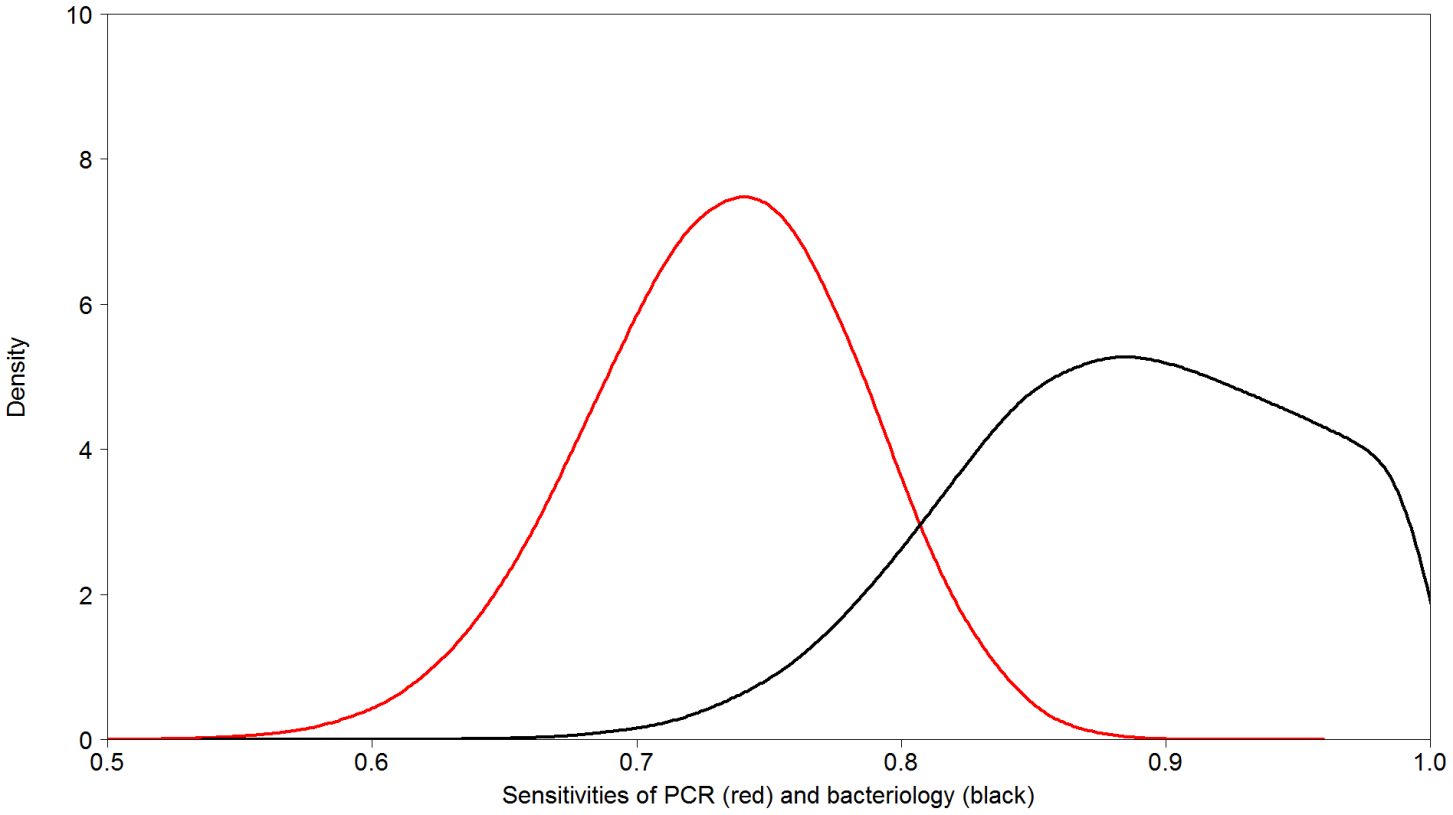
Diagnostic sensitivities of bacteriology and PCR. Diagnostic sensitivities culture and PCR for detection of *Brachyspira hyodysenteriae* infection in pigs, estimated by latent class analysis. Results are given in the form of posterior density distributions, and show that culture has a higher sensitivity than PCR (red  = PCR, black  =  bacteriology).

**Figure 2:**
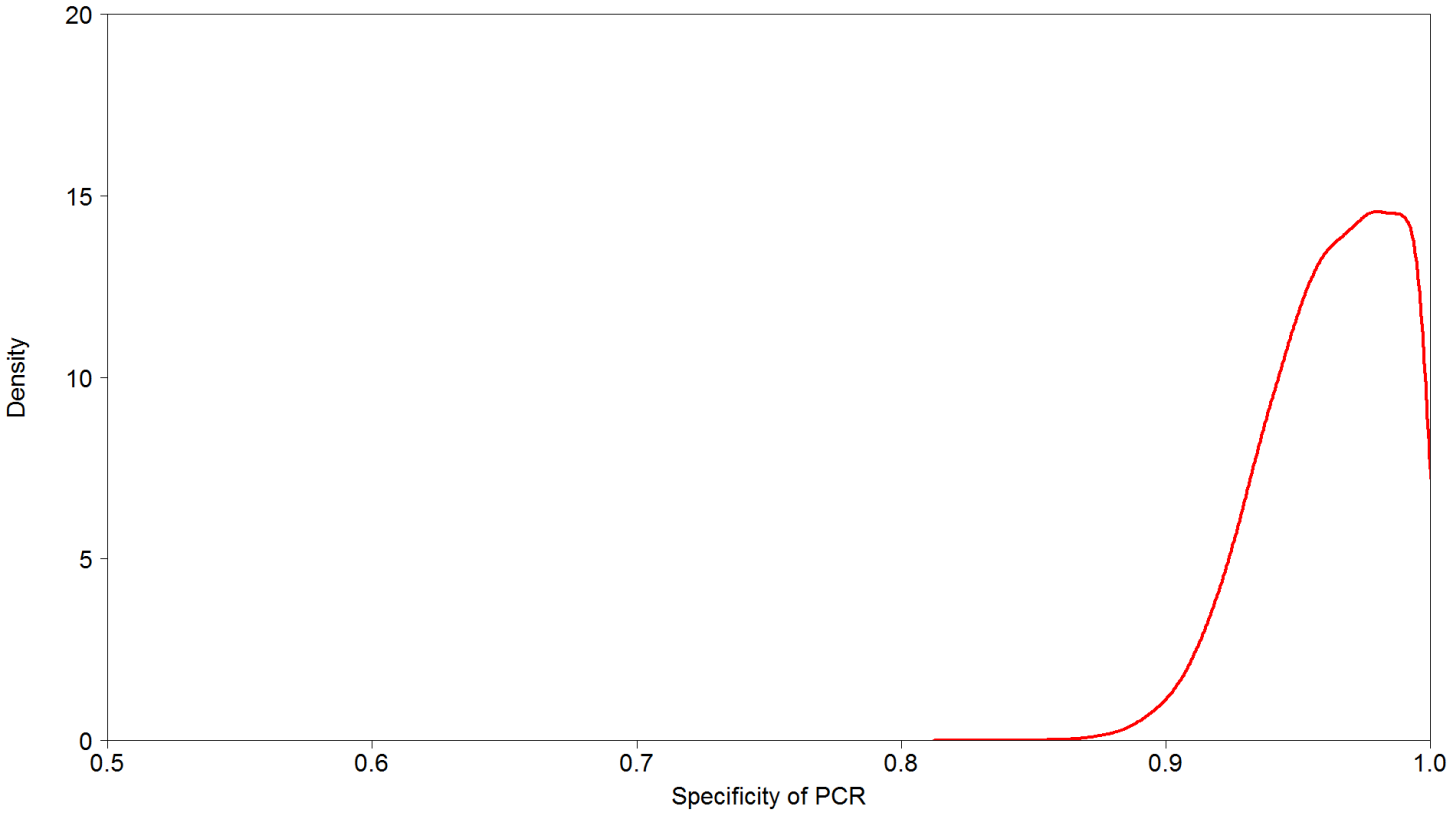
Diagnostic specificity of PCR. Diagnostic specificity of PCR for detection of *Brachyspira hyodysenteriae* infection in pigs, estimated by latent class analysis. Results are given in the form of a posterior density distribution.

Two latent class models, one with and one without a covariance term have been tested to obtain estimates for the parameters of interest. Based on DIC as a decision criterion (without test sensitivity covariance: 453.6 and with tests sensitivity covariance: 454.3), the model without any covariance structure between tests was chosen due to the principle of parsimony. Additionally, the inclusion of the covariance yielded similar values for the parameters of interest. Posterior means and corresponding 95% credibility intervals for all parameters of interest are shown in table 2. Posterior estimates for the sensitivity of the PCR and culture, and the specificity, are shown in figures 1 and 2. Four chains were run from different starting points (figures S1–S3), and after burn-in converged to a common distribution. Additionally, convergence was also confirmed by the Gelman-Rubin statistics. Utilising an uninformative beta prior for the specificity of culture instead of fixing it –based on expert opinion equal to 1- led to virtually unchanged estimates for the sensitivity of culture and the specificity of PCR, but to an increased sensitivity of PCR of 83% with a wider 95% CI (67.2,98.7). The estimate of the specificity culture was 94.6% with a 95% CI (90.9,99.7). A sensitivity analysis of different beta priors is presented in table S1 indicating a minor influence of the different beta priors compared to non-informative priors. A sensitivity analysis for the gamma priors modelling the shape parameters suggested that the model would not converge (MCMC sampler failure) with wider priors e.g. (0.01,0.01) or (0.0001,0.0001), but did so with (0.5,0.0005) (results are present in table S2).

**Table 2:** Estimates (posterior means) of diagnostic test accuracies for the diagnosis of *Brachyspira hyodysenteriae* by culture and PCR and their corresponding 95% credibility intervals resulting from a Bayesian latent class model.

Culture		PCR	
Sensitivity	Specificity	Sensitivity	Specificity
(95% CI)		(95% CI)	(95% CI)
88.6	1*	73.2	96.2
(74.9;99.3)		(62.3;82.9)	(90.9;99.8)

*Specificity of culture was fixed to 1.

## Discussion/Conclusions

By applying a Bayesian latent class approach to data from large-scale routine laboratory testing, robust estimates for diagnostic test accuracies were obtained. The benefit of the random effects model becomes evident when un-informative priors for the sensitivities and specificities of PCR and culture are considered. In the case of a classical Hui-Walter model Bayesian inferences based on a single population with two tests will be imprecise e.g. a non-identifiable situation [10]. A reason for the sampler failing in the sensitivity analysis of the gamma priors is potentially explained by the variable number of samples tested per herd (a small number of herds had very few animals).

As – strictly speaking – diagnostic test accuracies are population dependent characteristics [29] (as opposed to some fixed intrinsic value related only to the diagnostic test being used), there is a need to evaluate diagnostics tests in the population of interest. The Field Station for Epidemiology in Bakum receives samples from a significant number of swine herds in North-West Germany, thus representing the population in the centre of the German pig industry. The obtained estimates are not necessarily directly applicable to another population e.g. a surveillance program aiming at identifying subclinical cases shedding presumably low levels of spirochetes or to a population consisting only of severe clinical cases of SD.

This approach allowed estimation of the specificity of the PCR without the need for a gold standard reference test, which is not available for *B. hyodysenteriae*. The reasonably good sensitivity and high specificity of the PCR compared to cultural testing, together with financial advantages makes the PCR a valuable diagnostic tool in which large numbers of samples can be tested for *B. hyodysenteriae*. From the perspective of a routine laboratory, the greatest advantage of the PCR is to enable the rapid implementation of therapeutic measures and preventive programmes in pig farms intended to control SD. Using standard isolation methods, the detection of *B. hyodysenteriae* usually requires 5–7 days. Conversely, PCR results can be provided in 2 days at the longest. Beside this advantage for PCR, this method is lacking the possibility to detect novel pathogenic spirochetes such as those recently identified in North America associated with SD [30]. In case of suspicion of e.g. weakly beta hemolytic *Brachyspira spp.* culture could be the method of choice.

The main novelty of the Bayesian latent class approach utilized here lies in its use of random effects to model the complex population structure of food animals, i.e. by allowing different farms to have very different prevalences (resulting from within farm clustering of disease). This is practically important, as without such flexibility the results of any analyses (i.e. estimates of sensitivity and specificities) are likely to be highly misleading.

In risk factor studies, accounting for clustering is considered good, indeed arguably essential, statistical practice. Diagnostic test evaluation studies, for tests applied at a regional or national level, have identical issues which should be accounted for given the complexities of population structure in food animal production. Bayesian latent class approaches can be readily utilized for this purpose.

## Supporting Information

Figure S1 **Four simulated chains for the sensitivity of PCR.** To ensure numerical robustness of the estimated diagnostic sensitivity of PCR, multiple simulations were performed using a Bayesian latent class model. The posterior density estimates of the diagnostic sensitivities were compared across all four simulations to check that the results were similar. It can be seen that the curves (densities) are almost identical, therefore providing strong confidence in the results.
(TIFF)

Figure S2 **Four simulated chains for the sensitivity of culture.** To ensure numerical robustness of the estimated diagnostic sensitivity of culture, multiple simulations were performed using a Bayesian latent class model. The posterior density estimates of the diagnostic sensitivities were compared across all four simulations to check that the results were similar. It can be seen that the curves (densities) are almost identical, therefore providing strong confidence in the results.
(TIFF)

Figure S3 **Four simulated chains for the specificity of PCR.** To ensure numerical robustness of the estimated diagnostic specificity of PCR, multiple simulations were performed using a Bayesian latent class model. The posterior density estimates of the diagnostic sensitivities were compared across all four simulations to check that the results were similar. It can be seen that the curves (densities) are almost identical, therefore providing strong confidence in the results.
(TIFF)

Table S1 **Sensitivity analysis for different beta priors for test accuracies.**
(DOCX)

Table S2 **Sensitivity analysis with different priors for the gamma priors.**
(DOCX)

Code S1 **Code for the final model.**
(DOCX)
