# Supplementary figures and images for: Estimating Diagnostic Test Accuracies for Brachyspira hyodysenteriae Accounting for the Complexities of Population Structure in Food Animals
Source: PLoS One. 2014 Jun 6;9(6):e98534. doi: 10.1371/journal.pone.0098534 (PMC4048188; doi:10.1371/journal.pone.0098534)

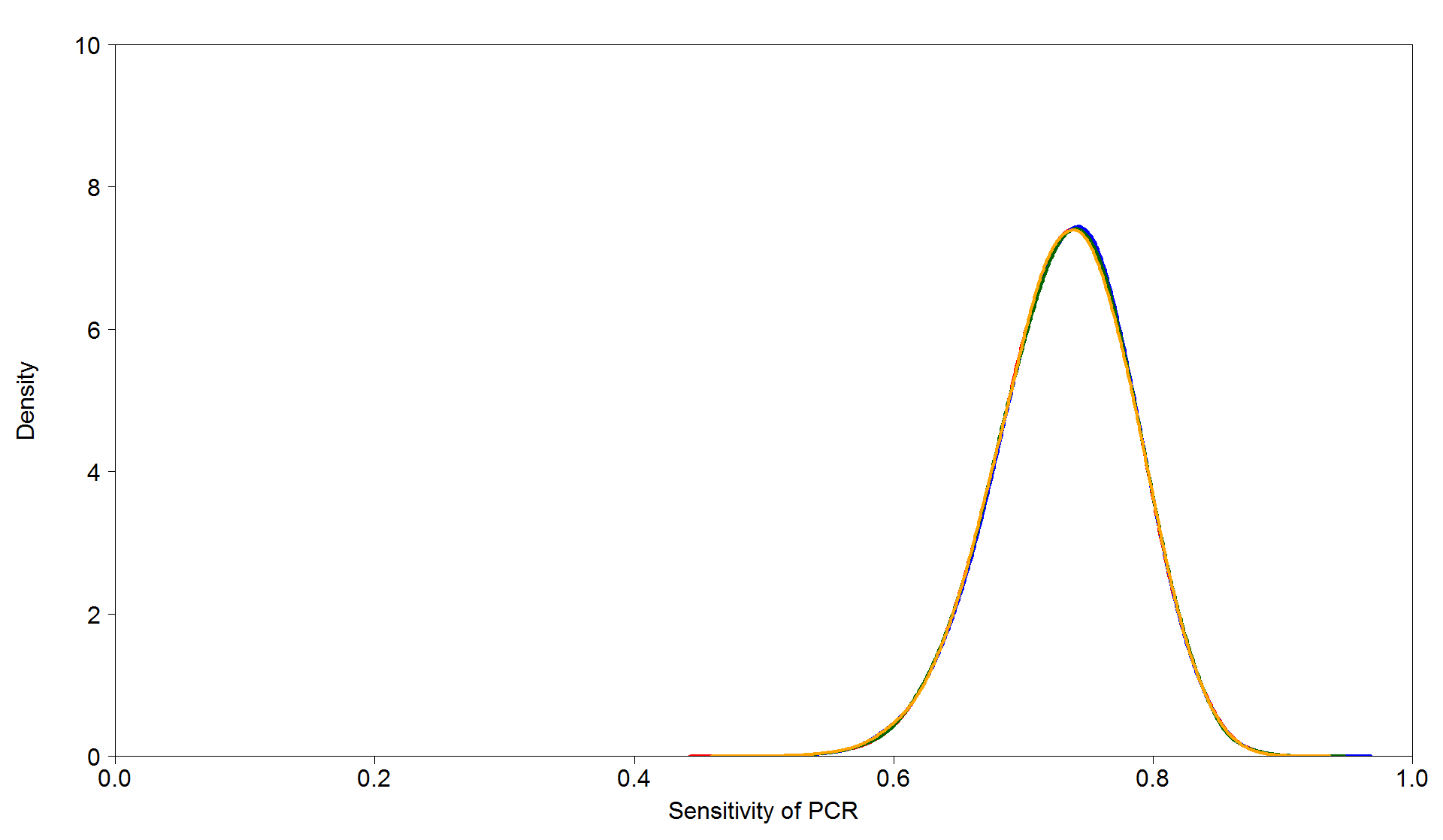

Supplement: Figure S1 — Four simulated chains for the sensitivity of PCR. To ensure numerical robustness of the estimated diagnostic sensitivity of PCR, multiple simulations were performed using a Bayesian latent class model. The posterior density estimates of the diagnostic sensitivities were compared across all four simulations to check that the results were similar. It can be seen that the curves (densities) are almost identical, therefore providing strong confidence in the results. (TIFF) [file pone.0098534.s001.tiff]

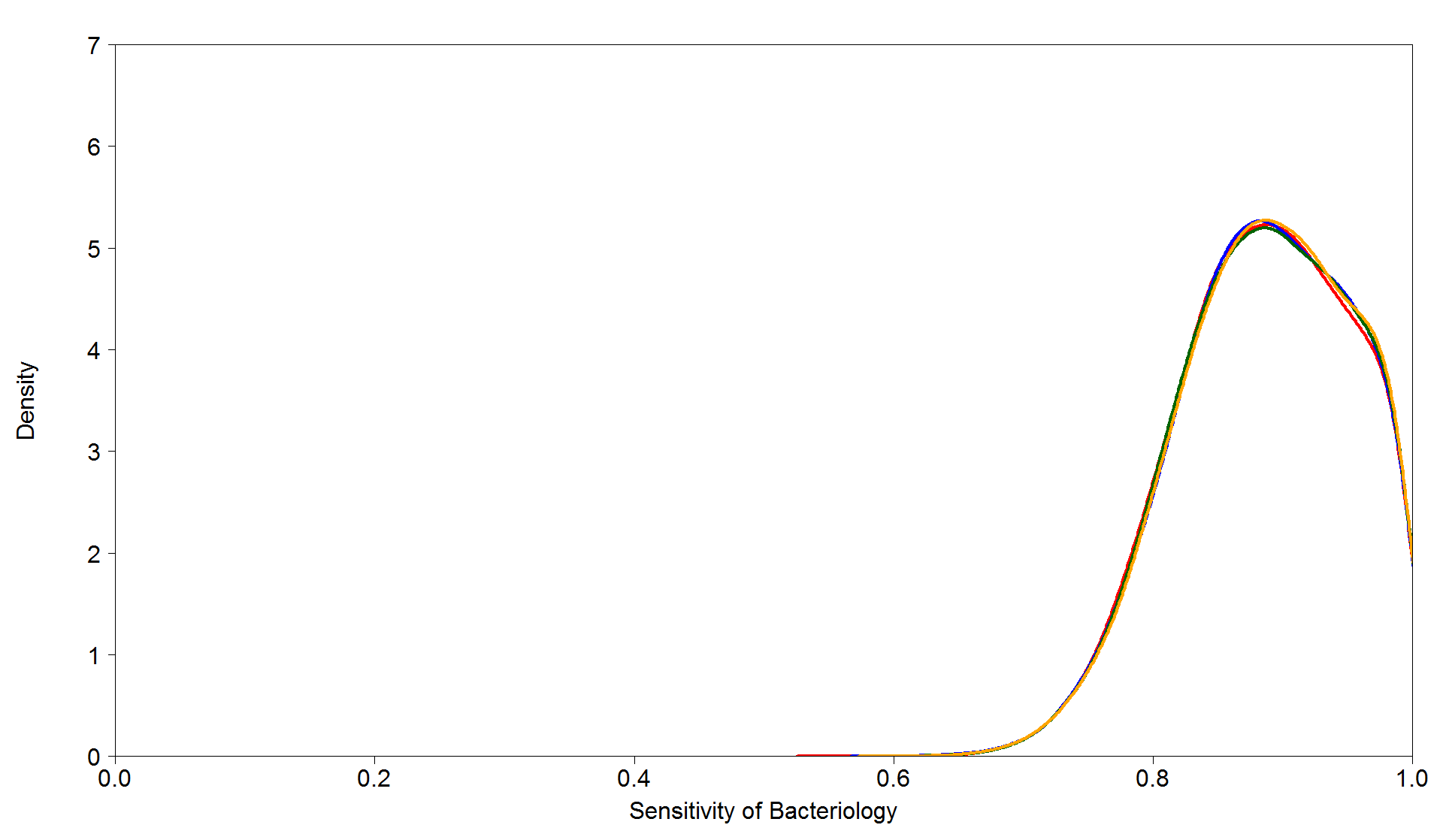

Supplement: Figure S2 — Four simulated chains for the sensitivity of culture. To ensure numerical robustness of the estimated diagnostic sensitivity of culture, multiple simulations were performed using a Bayesian latent class model. The posterior density estimates of the diagnostic sensitivities were compared across all four simulations to check that the results were similar. It can be seen that the curves (densities) are almost identical, therefore providing strong confidence in the results. (TIFF) [file pone.0098534.s002.tiff]

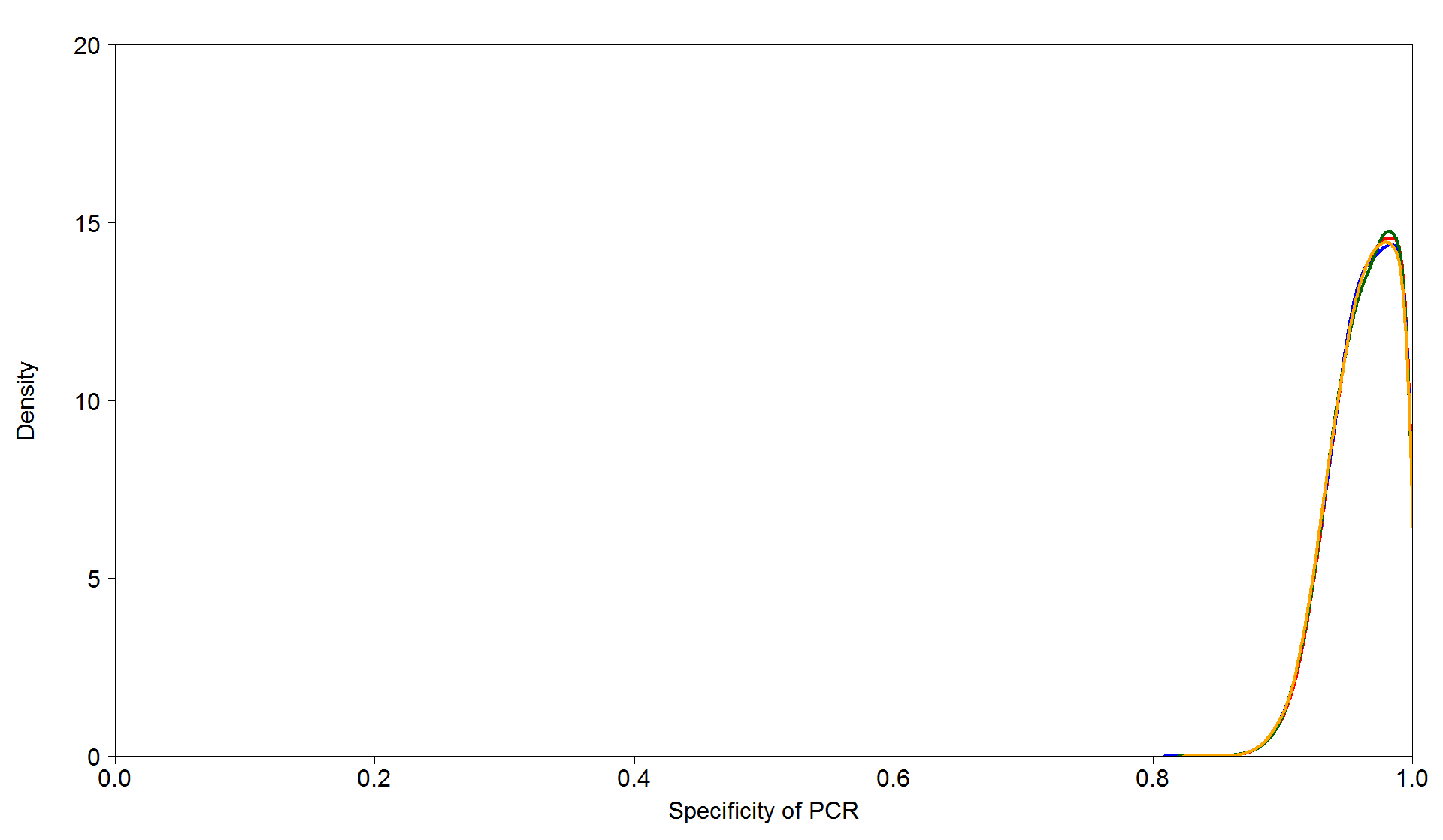

Supplement: Figure S3 — Four simulated chains for the specificity of PCR. To ensure numerical robustness of the estimated diagnostic specificity of PCR, multiple simulations were performed using a Bayesian latent class model. The posterior density estimates of the diagnostic sensitivities were compared across all four simulations to check that the results were similar. It can be seen that the curves (densities) are almost identical, therefore providing strong confidence in the results. (TIFF) [file pone.0098534.s003.tiff]
