# Supplementary material for: Estimating Diagnostic Test Accuracies for Brachyspira hyodysenteriae Accounting for the Complexities of Population Structure in Food Animals
Source: PLoS One. 2014 Jun 6;9(6):e98534. doi: 10.1371/journal.pone.0098534 (PMC4048188; doi:10.1371/journal.pone.0098534)
Supplement: Table S1 — Sensitivity analysis for different beta priors for test accuracies. (DOCX) [file pone.0098534.s004.docx]

Supplementary Table S1: Sensitivity analysis for different beta priors for test accuracies

|  | Priors for | | | | | |
| --- | --- | --- | --- | --- | --- | --- |
| Beta priors | s1 | | s2 | | c1 | |
| (4.8416,3.5611)*^1^ | s1 | 72.1 (61.7,81.3) | s1 | 72.6 (61.8,82.3) | s1 | 73.1 (62.3,82.6) |
|  | s2 | 88.4 (74.5,99.3) | s2 | 82.8 (71.5,93.3) | s2 | 93.3 (80.9,99.8) |
|  | c1 | 96.3 (90.9,99.8) | c1 | 97.6 (92.9,99.9) | c1 | 93.3 (88.3,97.4) |
|  | c2 | 1 (1,1) | c2 | 1 (1,1) | c2 | 1 (1,1) |
|  | | | | | | |
| (4.607,1.9017)*^2^ | s1 | 73.7 (63.1,82.9) | s1 | 73.1 (62.3,82.7) | s1 | 73.2 (62.5,82.8) |
|  | s2 | 89.1 (75.4,99.3) | s2 | 86.7 (74.4,97.4) | s2 | 90.9 (77.3,99.5) |
|  | c1 | 96.2 (90.8,99.8) | c1 | 96.8 (91.6,99.9) | c1 | 95 (89.9,99.1) |
|  | c2 | 1 (1,1) | c2 | 1 (1,1) | c2 | 1 (1,1) |
|  | | | | | | |
| (4.7719,1.1985)*^3^ | s1 | 74.4 (64,83.6) | s1 | 73.3 (62.6,82.7) | s1 | 73.2 (62.3,82.9) |
|  | s2 | 89 (75.8,99.3) | s2 | 89.5 (76.7,99.1) | s2 | 89 (75.4,99.3) |
|  | c1 | 96.2 (90.7,99.8) | c1 | 96 (90.7,99.8) | c1 | 96.1 (90.9,99.7) |
|  | c2 | 1 (1,1) | c2 | 1 (1,1) | c2 | 1 (1,1) |

s1=sensitivity PCR, s2=sensitivity culture, c1=specificity PCR

*^1^ Input information in BetaBuster 95% sure, that x is greater than 0.3 and mode at 0.6

*^2^ Input information in BetaBuster 95% sure, that x is greater than 0.4 and mode at 0.75

*^3^ Input information in BetaBuster 95% sure, that x is greater than 0.5 and mode at 0.95

Supplementary Table S2

Sensitivity analysis with different priors for the gamma priors*

| Prior for shape parameter | A  posterior mean (95% CI) | B  posterior mean (95% CI) |
| --- | --- | --- |
| [0.01,0.01] | Not converging | Not converging |
| [0.001,0.001] | Not converging | Not converging |
| [0.5,0.0005] | Se PCR 73.3 (62.5,82.7)  Sp PCR 96.1 (90.8,99.8)  Se Cu 89.3 (75.6,99.4) | Se PCR 73.1 (62.5, 82.8)  Sp PCR 96 (90.8,99,8)  Se Cu 89.2 (75.5,99.3) |

*choice of the gamma priors according to

Wakefield J, Best N, Waller L (2000) Bayesian approaches in disease mapping. In: Elliott P, Wakefield J, Best N, Briggs D, eds. Spatial Epidemiology. Methods and Applications. Oxford: Oxford University Press 104-127

Lawson A, Browne W, Vidal Rodeiro C (2003) Disease Mapping with WinBUGS and MLWin. Chichester: John Wiley & Sons
