# Supplementary material for: Estimating Diagnostic Test Accuracies for Brachyspira hyodysenteriae Accounting for the Complexities of Population Structure in Food Animals
Source: PLoS One. 2014 Jun 6;9(6):e98534. doi: 10.1371/journal.pone.0098534 (PMC4048188; doi:10.1371/journal.pone.0098534)
Supplement: Table S2 — Sensitivity analysis with different priors for the gamma priors. (DOCX) [file pone.0098534.s005.docx]

Supplementary Table S2

Sensitivity analysis with different priors for the gamma priors*

| Prior for shape parameter | A  posterior mean (95% CI) | B  posterior mean (95% CI) |
| --- | --- | --- |
| [0.01,0.01] | Not converging | Not converging |
| [0.001,0.001] | Not converging | Not converging |
| [0.5,0.0005] | Se PCR 73.3 (62.5,82.7)  Sp PCR 96.1 (90.8,99.8)  Se Cu 89.3 (75.6,99.4) | Se PCR 73.1 (62.5, 82.8)  Sp PCR 96 (90.8,99,8)  Se Cu 89.2 (75.5,99.3) |

*choice of the gamma priors according to

Wakefield J, Best N, Waller L (2000) Bayesian approaches in disease mapping. In: Elliott P, Wakefield J, Best N, Briggs D, eds. Spatial Epidemiology. Methods and Applications. Oxford: Oxford University Press 104-127

Lawson A, Browne W, Vidal Rodeiro C (2003) Disease Mapping with WinBUGS and MLWin. Chichester: John Wiley & Sons
