# Supplementary material for: Estimating Diagnostic Test Accuracies for Brachyspira hyodysenteriae Accounting for the Complexities of Population Structure in Food Animals
Source: PLoS One. 2014 Jun 6;9(6):e98534. doi: 10.1371/journal.pone.0098534 (PMC4048188; doi:10.1371/journal.pone.0098534)
Supplement: Code S1 — Code for the final model. (DOCX) [file pone.0098534.s006.docx]

Model description

*♯* *Each row in the data is a tested sample and the columns are the different test results, herd identifier*

*♯ p represents the individual data points, q the different test result combinations from q[i,1] as ++ to q[i,4] as --, pr the prevalence, rv to model the prevalence on each individual farm as a random effect,s_1_ the sensitivity of PCR, s2 the sensitivity of bacteriology and c_1_ the specificity of PCR, covs12 is the conditional dependency between the two test sensitivities, m.brall represents the name of the data set*

##############################################################################################

var pr[N],p[N],q[N,4],checks[N,8],rv[M]; #tells jags the size of vectors

♯ *To define the individual parameters within the likelihood function. To model the 4 probabilities following a multinomial distribution with a loop for each observation.*

model {

for(j in 1:M){rv[j]~dbeta(shape1[1],shape2[1]);}

for(i in 1:N){#for each observation in the dataset

q[i,1] <-p[i]*(s1*s2+covs12) +(1-p[i])*((1-c1)*(1-c2)+covc12);

q[i,2] <-p[i]*(s1*(1-s2)-covs12) +(1-p[i])*((1-c1)*c2-covc12);

q[i,3] <-p[i]*((1-s1)*s2-covs12) +(1-p[i])*(c1*(1-c2)-covc12);

q[i,4]<-p[i]*((1-s1)*(1-s2)+covs12)+(1-p[i])*(c1*c2+covc12);

### *♯ to describe which observation is on which farm*

p[i]<-rv[m.brall[i,2]];

*♯ Error checking since (0,1) bounds could be exceeded which is not allowed as dealing with probabilities (if so, the parameter combination is allocated a vanishingly small likelihood value)*

checks[i,1]<- s1*s2+covs12;

checks[i,2]<- (1-c1)*(1-c2)+covc12;

checks[i,3]<- s1*(1-s2)-covs12;

checks[i,4]<- (1-c1)*c2-covc12;

checks[i,5]<- (1-s1)*s2-covs12;

checks[i,6]<- c1*(1-c2)-covc12;

checks[i,7]<- (1-s1)*(1-s2)+covs12;

checks[i,8]<- c1*c2+covc12;

valid[i]<-step(1-q[i,1])*step(q[i,1])*

step(1-q[i,2])*step(q[i,2])*

step(1-q[i,3])*step(q[i,3])*

step(1-q[i,4])*step(q[i,4])*

step(1-checks[i,1])*step(checks[i,1])*

step(1-checks[i,2])*step(checks[i,2])*

step(1-checks[i,3])*step(checks[i,3])*

step(1-checks[i,4])*step(checks[i,4])*

step(1-checks[i,5])*step(checks[i,5])*

step(1-checks[i,6])*step(checks[i,6])*

step(1-checks[i,7])*step(checks[i,7])*

step(1-checks[i,8])*step(checks[i,8]);

*♯ Define/compute the contribution to the likelihood for the i^th^ observation*

L[i]<- equals(valid[i],1)*(

equals(m.brall[i,4],1)*equals(m.brall[i,8],1)*q[i,1]

+ equals(m.brall[i,4],1)*equals(m.brall[i,8],0)*q[i,2]

+ equals(m.brall[i,4],0)*equals(m.brall[i,8],1)*q[i,3]

+ equals(m.brall[i,4],0)*equals(m.brall[i,8],0)*q[i,4]

) +(1-equals(valid[i],1)) *(1e-14);

pr[i] <- L[i] / 1;

ones[i] ~ dbern(pr[i]);

}

*♯ Define priors (which may be posterior distributions or fixed values)*

s1~dbeta(1,1);

s2~dbeta(1,1);

c1~dbeta(1,1);

c2<-1;

for(i in 1:1){shape1[i]~dgamma(5,0.01);shape2[i]~dgamma(5,0.01);}

covs12<-0;

covc12<-0;

logL<-sum(log(p[1:N]));

}
